# A single session of education for a patient with negative beliefs about low back pain: A case report of 16-month follow-up

**DOI:** 10.25122/jml-2022-0248

**Published:** 2023-02

**Authors:** Ali Muteb Alshami

**Affiliations:** 1 Department of Physical Therapy, Imam Abdulrahman Bin Faisal University, Dammam, Saudi Arabia

**Keywords:** case report, long-term, musculoskeletal, physical therapy, rehabilitation, CLBP – chronic low back pain, FABQ – Fear Avoidance Belief Questionnaire, LBP – low back pain, MRI – magnetic resonance imaging, NPRS – numeric pain rating scale, PSFS – patient specific functional scale, RCT – randomized controlled trial, SBST – Keele STarT Back Screening Tool

## Abstract

The effectiveness of education in patients with low back pain (LBP) remains controversial and inconclusive. This case report describes the long-term effects of a single educational session on the rehabilitation of a patient with chronic LBP (CLBP). A 57-year-old woman presented with the main complaint of LBP and inability to prostrate for several years. The intervention consisted of a single session of patient-specific education that targeted negative cognitive beliefs. This education included instructions about the obtained findings, spinal anatomy, patient reassurance, the relationship between imaging findings and patient symptoms, proposed treatment, and a home exercise program. The patient was able to independently complete the prostration task immediately after the session without pain. This improvement was maintained for at least 16 months, as demonstrated by the Numeric Pain Rate Scale, Patient-Specific Functional Scale, Fear Avoidance Belief Questionnaire, and the Keele STarT Back Screening Tool. In conclusion, a single session of patient-specific education was effective, both immediately and over the long term, in addressing pain and function in patients with CLBP.

## INTRODUCTION

Low back pain (LBP) is the single principal cause of disability in 160 countries worldwide. Clinical practice guidelines for LBP recommend physical and psychosocial therapies with less focus on pharmacological and surgical treatments. These therapies include methods of patient education such as traditional biomedical education, cognitive behavioral therapy, and pain neuroscience education, typically delivered by a trained therapist over several sessions [1].

Although patient education has been recommended as a first-line treatment for acute and chronic LBP, its effectiveness in patients with LBP is conflicting and inconclusive. For example, a recent systematic review concluded that patient education improves pain, disability, and quality of life in patients with LBP. Out of five studies reviewed, only two showed significant improvement after the education program [2]. A previous systematic review included 13 randomized controlled trials (RCTs) and concluded that education programs were not effective in reducing pain, disability, and quality of life in patients with LBP [3].

More studies are needed to provide evidence of the effectiveness of education in patients with LBP, owing to a lack of evidence and a limited number of RCTs [2]. Although several education methods can be provided to patients with LBP, no single method has been found to be superior. In addition, these methods may have various limitations, such as time, cost, and availability [1]. Studies on the long-term effects of a single education session on patients with CLBP are lacking. Lower-intensity treatment options with a single session may be sufficient for a group of patients. Furthermore, a single-session intervention may be no less effective than multiple long-term sessions of intervention that have several obstacles, such as limited patient access, time, costs, and therapist availability [1]. Therefore, the current study aimed to describe the outcomes of a patient with persistent LBP who did not respond to previous conservative interventions. However, she responded favorably over the long term to a single educational session.

## CASE REPORT

**Figure 1:**
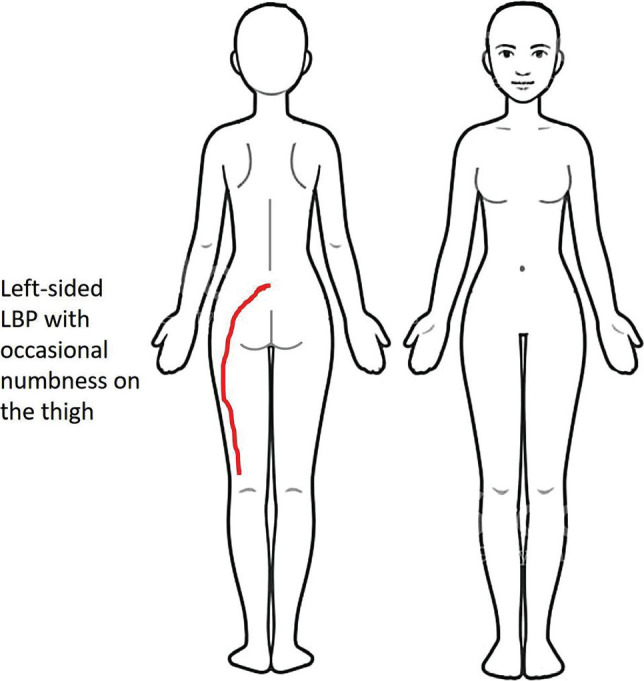
Body chart of the patient showing the main complaint of left-sided low back pain and occasional numbness of the left thigh.

The patient was a 57-year-old woman who had been unemployed for the last ten years and had worked as a teacher for 5–6 years. She presented to the clinic for consultation regarding her chief complaint of left-sided LBP with occasional numbness in the lateral left thigh (Figure 1). This pain prevented her from sitting on her left buttock or prostration. Prostration involves the position in which the person kneels and bows until the forehead, nose, and palms of the hands contact the ground. She reported that the initial pain started approximately three years ago at home while lying on her left side in bed. The patient attempted several medications and physical therapy techniques, but the results were unsatisfactory. The patient had a previous diagnosis of benign multiple sclerosis 18 years ago, with full recovery 2 years later. She was advised by her physician to take Neurontin once daily for five months to prevent seizures that may be associated with multiple sclerosis. This medication was discontinued before the time of the session. She was diagnosed with bilateral knee osteoarthritis 10 years ago.

The patient described the intensity of her current LBP as 3/10 on the numeric pain rating scale (NPRS). Her numbness on the lateral thigh was described as a "cotton feeling" (Figure 1). The patient had not been able to prostrate for the last 10 years. She believed that the reasons for not being able to prostrate were knee osteoarthritis and recent magnetic resonance imaging (MRI) results showing that the discs may "bulge with doing this movement". She was told by a physician not to prostrate because of knee osteoarthritis. Recently, another physician advised her not to prostrate because this movement could worsen the lumbar disc bulge.

### Assessments

Initial radiography revealed spondylotic changes in the form of marginal osteophytes detected at L2, L3, L4, and L5. MRI showed diffuse disc bulges at L2-3, L3-4, L4-5, and L5-S1, with narrowing of the neural foramina and abutting of both exiting nerves at L4-5. Multiple facet joint arthropathies and ligamentum flavum thickening were also observed.

#### Physical examination and tests

The author, a consultant physical therapist with more than 20 years of experience in the management of musculoskeletal pain disorders, conducted the clinical examinations.

#### Initial observation and posture

The patient walked independently in the clinic and did not appear to experience pain. Formal observation of posture was not performed because of the priority of other tests and its poor relationship with nonspecific LBP [4].

#### Movement testing

Examination of the lumbar spine while standing included active movement testing for forward bending (flexion), extension, lateral flexion, and rotation on both sides with/without overpressure [4]. All movements were within the normal range, except for flexion, which was within the 75% range and reproduced severe LBP at the end of the range and with a return from flexion.

#### Neurodynamic provocation test

Straight leg raise was passively tested on both legs in the supine position. The hips were flexed to 80° and reproduced tightness behind the thigh after performing ankle dorsiflexion as a distal sensitizing movement. The test was considered negative because it did not reproduce the symptoms [4].

#### Manual testing

In the prone position, the central posterior-anterior vertebral pressures from L1 to S1 reproduced moderate pain over L4 and L5. Left unilateral posterior-anterior vertebral pressure reproduced moderate pain over the L4/L5 and L5/S1 facet joints. The test did not reproduce the thigh numbness. Abnormal vertebral motion is moderately helpful for predicting responses to particular conservative treatments [4].

#### Knee testing

Since the patient reported that she had been diagnosed with knee osteoarthritis over the last 10 years, the author decided to quickly screen her hips and knees. Manual muscle testing for hip and knee extension was 4-/5 and 4/5, respectively. No range of motion deficits was found in any hip or knee movement. Mild tightness was reported by the patient in the anterior thighs at the end range of knee flexion during the prone knee-bend test.

#### Self-report and outcome measures

NPRS was used to assess pain intensity at rest [4]. In addition, the patient was asked to rate up to three activities that were challenging to perform or could not be accomplished due to LBP by completing the Patient Specific Functional Scale (PSFS) [5]. The Fear Avoidance Belief Questionnaire (FABQ) was used to evaluate patients' perceived fear of movement due to the presence of LBP with regard to predicting physical activity (FABQ-PA) and work loss (FABQ-W) [6]. Moreover, the Keele STarT back screening tool (SBST) was used to screen prognostic indicators for persistent disabling LBP with categories of low, medium, or high risk [7].

Table 1 shows the self-reported and outcome measures, including the minimal clinically important difference (MCID) and minimal detectable change (MDC). At the initial session, the patient had moderate pain and high fear avoidance beliefs that adversely affected physical activity and was classified as having a high risk of poor outcomes and developing persistent LBP.

**Table 1:** Self-reported outcome measurements during the follow-up period.

Outcome	Baseline	16 months	Test-retest reliability	MCID/MDC
**NPRS (0–10)**	7–8 (at rest)	0 (at rest) 3 (after 5 hours of standing)	ICC=0.72	2 [8]
**PSFS (0–10)**			ICC=0.92 [5]	1.4 [5]
Bending in prayer	8	10	-	-
Sitting	5	10	-	-
**FABQ-W (0–42)**	30	1	ICC=0.95 [6]	5.95 [6]
**FABQ-PA (0–24)**	24	4	ICC=0.90 [6]	3.69 [6]
**SBST**			ICC=0.89 [7]	N/A
Total (0–9)	8	0	-	-
Subscale (0–5)	5	0	-	-

NPRS – Numerical Pain Rating Scale (0-10); PSFS – Patient Specific Functional Scale; FABQ – Fear-Avoidance Beliefs Questionnaire (W – Work subscale; PA – Physical Activity subscale); SBST – Keele STarT Back Screening Tool; MCID – minimal clinically important difference; MDC – minimal detectable change.

### Diagnosis, evaluation, and clinical reasoning

The author believed that the patient's severe LBP and inability to prostrate for the last 10 years were primarily related to cognitive function associated with negative beliefs about her condition. The patient's impairments (such as pain on lumbar flexion testing, pain in the lower lumbar spine with pressure, and mild tightness and weakness of the quadriceps) did not seem to be the main cause of the patient's symptoms and functional disability. Active movement testing of the lumbar spine and knees revealed unremarkable results. All movements of the lumbar spine were within normal limits except for mild range limitation and pain with flexion. The patient showed above-average muscle strength and complete knee range of motion. In addition, the patient visited different clinicians and attempted several treatments, but with unsatisfactory results. Moreover, the patient had high FABQ and SBST scores.

### Intervention

Based on the clinical examination and reasoning process of this patient, the author explained the findings, suspected disorder, and proposed treatment to the patient. The patient was instructed to perform the following exercises once per day at home for 2 sets of 15 repetitions: active straight leg raise in the supine position and active flexion in the prone position. Moreover, as the patient's daughter was a physical therapist, she was instructed to perform lumbar spine mobilization, which consisted of central and left unilateral posterior-anterior pressure (grade III) on the lower lumbar spine with three sets of 30 repetitions, once daily.

The patient was mainly concerned about the MRI findings of the lumbar spine and the disc bulge. The patient was unable to prostrate for several years due to her negative belief about the relationship between the bulging disc in her lumbar spine and knee osteoarthritis and her symptoms. Patient education was the main goal of the session. Patient-specific education comprised information about the obtained findings, spinal anatomy, patient reassurance, the relationship between the MRI findings and patient symptoms, the proposed treatment, and a home exercise program. At the end of the session, the patient was asked to perform prostration. The session lasted approximately 1.5 hours, including the patient's history, examination, and education.

### Outcomes

Interestingly, after the session, the patient performed prostration with no pain in the lower back, left thigh, or knee. Accordingly, the patient was instructed to immediately pray normally at home, which included prostration and no need to use a chair. Although there were no follow-up sessions, the author contacted the patient five days after the initial session to ask about her progression. The patient reported that she had been performing a prostration task normally without pain since the initial session. To examine the long-term effect of this session, the patient was asked to complete self-report measures after 16 months. Clinically meaningful improvements were observed in all outcomes: NPRS=0/10 (at rest) and 3/10 (after 5 hours of standing), PSFS=10/10 (prostration and sitting), FABQ-W=1/42, FABQ-PA=4/24, and SBST=0/9 (total) and 0/5 (subscale) (Table 1). The patient reported that the home exercise program was performed for only one month and that lumbar spine mobilization was performed only twice. No adverse or unanticipated events were reported. The patient declared that she did not use any other interventions throughout the study.

## DISCUSSION

The patient in this case report, who complained of recalcitrant LBP and a long history of bilateral knee osteoarthritis, was able to independently prostrate without pain immediately after a single session of patient-specific education. Interestingly, this rapid and clinically meaningful improvement in pain and function lasted at least 16 months. Improvements in pain intensity (NPRS), related disability (PSFS), perceived fear of movement for work (FABQ-W), and physical activity (FABQ-PA) exceeded the MCID/MDC of 2 cm [8], 1.4 points [5], 5.95 points, and 3.69 points [6], respectively. The patient was at high risk for developing persistent LBP and activity limitation (SBST) at the initial session, which improved to a low risk for at least 16 months.

A 1.5-hour session of education was provided by a physical therapist who was not specially trained in psychology. The education was not structured; it was patient-specific and included information about the obtained findings, spine anatomy, patient reassurance, the relationship between MRI findings and patient symptoms, proposed treatment, and home exercise programs. This type of education aimed to target patients' negative cognitive beliefs about their condition. It is recommended that patient education alone may be insufficient and best combined with other modalities, such as exercises [2]. Typically, patient education methods are time-consuming and require psychologists or specially trained therapists [1]. However, the current case report demonstrated that a short single session of patient-specific education resulted in an immediate and long-term clinically meaningful change in pain and related disability.

The clinical relevance of this case report is the importance of patient-specific education in the rehabilitation of patients with chronic musculoskeletal pain, especially LBP and knee osteoarthritis. This case report provides preliminary evidence regarding the long-term effects of a single session of patient-specific education. However, future RCTs are needed to further examine the clinical cost-effectiveness of this educational method.

A limitation of this case report was that the author did not use Waddell's sign to classify this patient with negative beliefs about her LBP. However, a pilot study suggested that Waddell's "non-organic signs" is questionable and may not exclusively be non-organic tests [9]. Inter-observer reliability of the signs ranged from fair (K=0.33) to good (K=0.74) and was moderate (K=0.48–0.49) for the overall Waddell score. Intra-observer reliability varied from moderate (K=0.43) to very good (K=0.84) for the signs and good (K=0.65–0.68) for the overall Waddell score. Internal consistency was good for both the categories (K=0.65–0.72) and the signs (K=0.71–0.78) [10].

## CONCLUSION

With a single 1.5-hour session of patient-specific education, the patient demonstrated an immediate return to a pain-free prostration task and improvements in functional status that lasted 16 months. Further RCTs are needed to determine the cost-effectiveness of single or minimal sessions in patients with chronic pain syndrome.

## Data Availability

Further data is available from the corresponding author on reasonable request.
